# SVA insertion in X-linked Dystonia Parkinsonism alters histone H3 acetylation associated with *TAF1* gene

**DOI:** 10.1371/journal.pone.0243655

**Published:** 2020-12-14

**Authors:** Tiziana Petrozziello, Amanda M. Dios, Kaly A. Mueller, Christine A. Vaine, William T. Hendriks, Kelly E. Glajch, Alexandra N. Mills, Kotchaphorn Mangkalaphiban, Ellen B. Penney, Naoto Ito, Cara Fernandez-Cerado, Gierold Paul A. Legarda, M. Salvie Velasco-Andrada, Patrick J. Acuña, Mark A. Ang, Edwin L. Muñoz, Cid Czarina E. Diesta, Regina Macalintal-Canlas, Geraldine Acuña, Nutan Sharma, Laurie J. Ozelius, D. Cristopher Bragg, Ghazaleh Sadri-Vakili

**Affiliations:** 1 NeuroEpigenetics Laboratory, Healey Center for ALS at Mass General, Massachusetts General Hospital, Boston, Massachusetts, United States of America; 2 The Collaborative Center for X-linked Dystonia-Parkinsonism, Department of Neurology, Massachusetts General Hospital, Boston, Massachusetts, United States of America; 3 Sunshine Care Foundation, Roxas City, Capiz, Philippines; 4 Department of Pathology, College of Medicine, University of the Philippines, Manila, Philippines; 5 Department of Neurosciences, Makati Medical Center, Makati City, Philippines; Barts and The London School of Medicine and Dentistry Blizard Institute, UNITED KINGDOM

## Abstract

X-linked Dystonia-Parkinsonism (XDP) is a neurodegenerative disease linked to an insertion of a SINE-VNTR-Alu (SVA)-type retrotransposon within an intron of *TAF1*. This SVA insertion induces aberrant *TAF1* splicing and partial intron retention, thereby decreasing levels of the full-length transcript. Here we sought to determine if these altered transcriptional dynamics caused by the SVA are also accompanied by local changes in histone acetylation, given that these modifications influence gene expression. Because TAF1 protein may itself exhibit histone acetyltransferase activity, we also examined whether decreased TAF1 expression in XDP cell lines and post-mortem brain affects global levels of acetylated histone H3 (AcH3). The results demonstrate that total AcH3 are not altered in XDP post-mortem prefrontal cortex or cell lines. We also did not detect local differences in AcH3 associated with *TAF1* exons or intronic sites flanking the SVA insertion. There was, however, a decrease in AcH3 association with the exon immediately proximal to the intronic SVA, and this decrease was normalized by CRISPR/Cas-excision of the SVA. Collectively, these data suggest that the SVA insertion alters histone status in this region, which may contribute to the dysregulation of *TAF1* expression.

## Introduction

X-Linked Dystonia-Parkinsonism (XDP) is a progressive neurodegenerative movement disorder endemic to the island of Panay in the Philippines [1–3] that is characterized by the loss of medium spiny neurons in the striatum [2, 4]. Previous studies mapped the genetic XDP causal locus to *TAF1* [5, 6] which encodes TATA Binding Protein (TBP)-Associated Factor-1 (TAF1), the largest component of the multi-subunit TFIID complex involved in RNA polymerase II-mediated transcription [7–10]. In these analyses, all XDP individuals appeared to share a common haplotype consisting of seven sequence variants clustered in and around *TAF1*, specifically a 48-base pair (48-bp) deletion, five disease-specific single nucleotide changes (designated in the literature as DSC-1, -2, -3, -10, and -12), and an intronic SINE-VNTR-Alu (SVA)-type retrotransposon insertion [5, 6]. Recent studies have since expanded the shared haplotype to encompass thirteen XDP-specific variants, including the original known markers, that fall within a genomic segment exclusive to *TAF1* [11]. Of these disease-related variants, only the SVA has thus far been linked directly to clinical disease manifestation. Its 5’ end contains a repeated hexamer, (CCCTCT)_n_, the length of which is polymorphic among XDP patients and is inversely correlated with age at disease onset [12, 13]. Nevertheless, the possibility that any of the other variants within the haplotype may also contribute to pathogenesis is not excluded.

Alterations in *TAF1* transcription have been described in XDP fibroblasts, induced pluripotent stem cells (iPSCs) and their neural derivatives, post-mortem brain tissue, and blood [6, 11, 14–16] with some evidence that lower *TAF1* levels may be inversely correlated to the length of the hexameric repeat within the SVA [13]. Furthermore, our group recently described additional transcriptional abnormalities in XDP neural stem cells occurring around the large *TAF1* intron in which the SVA is inserted, specifically: (1) multiple aberrant splicing events terminating within *TAF1* intron 32 immediately proximal to the SVA insertion, the most abundant of which was annotated as transcript *TAF1-32i*, (2) increased partial intron retention (IR) incorporating a proximal segment of intron 32 sequence within mature *TAF1* mRNA, and (3) decreased exon usage immediately 3’ to intron 32 [11]. Importantly, the excision of the SVA by CRISPR/Cas9-mediated genome editing rescued all of these defects and normalized expression of the full-length *TAF1* transcript [11]. Other studies have also reported that SVA excision in XDP-derived iPSCs increases *TAF1* expression [16]. Together, these findings strongly suggest that the decreased *TAF1* expression consistently detected in XDP patients may be caused by aberrant transcriptional dynamics within intron 32 and flanking exons as a result of the SVA insertion. However, the precise mechanisms involved are yet to be elucidated.

Aberrant splicing and intron retention can interfere with the local chromatin landscape [17], thus suggesting that alteration in *TAF1* transcription may be due to epigenetic modifications. Similarly, retrotransposons, including long interspersed nuclear elements (LINEs) and Alu elements, are associated with epigenetic modifications, such as increases in DNA methylation and alterations in histone acetylation and methylation [18–24]. In general, SVAs are repressed by DNA methylation of their cytosine and guanine rich sites [25], as well as by repressive histone marks such as methylation [26–28]. Furthermore, several lines of evidence suggest that while silencing SVA insertions may decrease gene expression, the loss of epigenetic repression at these sites leads to retrotransposon expression [19], and SVAs may indirectly regulate permissive histone marks such as acetylation by promoting silencing [29]. Additionally, TAF1 protein activity has been linked to histone acetylation, specifically, the C-terminus domain contains a double bromodomain which is able to recognize acetylated histones [30]. Moreover, it was suggested that TAF1 possesses histone acetyltransferase (HAT) activity and thereby could regulate histone acetylation and gene expression [30–35]. Histone acetylation, finely regulated by HAT and histone deacetylases (HDAC) activity [36–38], consists of the addition of acetyl groups on histone tails decreasing the positive charge leading to decondensed chromatin and increased transcription [39–44]. Given that retrotransposons can regulate gene expression [45–52] and are in turn regulated by epigenetic modifications [18–23], it is therefore possible that alterations in permissive epigenetic marks such as histone acetylation may regulate *TAF1* expression in XDP.

Here, we assessed alterations in histone H3 acetylation in human post-mortem prefrontal cortex (PFC), a region characterized by relatively healthy neurons that are part of the striatal-cortical circuit, and fibroblasts derived from XDP patients and unaffected family members. Furthermore, we assessed acetylated histone association with sites within the *TAF1* gene in both XDP-derived fibroblasts and neuronal stem cells (NSCs). Lastly, we determined whether removing the SVA could reverse alterations in histone acetylation at specific *TAF1* gene loci in NSCs derived from XDP and controls.

## Methods

All methods were carried out in accordance with the guidelines and regulations of Massachusetts General Hospital and approved by the Massachusetts General Hospital licensing committees.

### Human tissue samples

All procedures related to the collection, processing, and the use of XDP patient post-mortem brain samples were approved by Institutional Review Boards (IRB) at Makati Medical Center (Makati City, Philippines; protocol MMCIRB 2017–134) and Massachusetts General Hospital (MGH; Boston, USA; protocol 2016p-000427). XDP patients were informed about the option of post-mortem brain donation by their movement disorder neurologist or by their genetic counselor, clinical care, and seminars offered by the Sunshine Care Foundation (Roxas City, Capiz Philippines). Individuals interested in participating in brain donations were afforded with additional information. Informed consent for post-mortem brain donation was acquired from all participants. Final consent was obtained from next of kin after family members or health care providers contacted the brain bank at the time of death. Brain bank staff immediately traveled to the local funeral home to perform an autopsy limited to brain. Brains were placed in an ice water bath and transported to the brain bank facilities for processing following removal. For each brain, one hemisphere was cut into 16 standard coronal sections and frozen between Teflon coated cold metal plates on dry ice and the opposite hemisphere was fixed in formalin. Here, fresh frozen PFC was dissected from the coronal sections of 11 XDP patient brains. Control tissue from 3 PFC was provided by the Massachusetts Alzheimer’s Disease Research Center (ADRC) with approval from the Massachusetts General Hospital IRB (1999p009556). Clinical information on the tissues used in this study are reported in Table 1.

**Table 1:** Human post-mortem prefrontal cortex from XDP and control subjects.

	Number of subjects	Sex	Age of onset	Age of death	Repeat size	PMI
**Control**	3	M	N/A	60–92	N/A	14–23
**XDP**	11	M	33–58	41–67	34–54	16–36

### Human fibroblast cultures

Human fibroblasts were provided by the MGH Collaborative Center for X-linked Dystonia Parkinsonism. The clinical characteristics of donor subjects, confirmation of XDP genotype, and derivation of fibroblasts were previously described [11, 15, 53]. In total we assessed histone AcH3 levels in fibroblasts derived from 6 XDP patients and 10 unaffected family members as well as histone AcH3 association across *TAF1* in fibroblasts derived from 7 XDP patients and 14 unaffected family members. All fibroblasts were cultured in Dulbecco’s Modified Eagle’s Medium (DMEM) supplemented with 20% fetal bovine serum (FBS) and 1X Penicillin/Streptomycin/L-glutamine and grown in an incubator at 37°C, 5% CO_2_.

### Neuronal stem cells (NSCs) cultures

NSCs were provided by the MGH Collaborative Center for X-linked Dystonia Parkinsonism. Induced pluripotent stem cells reprogramming, neuronal differentiation and CRISPR/Cas9 nuclease-mediated genome editing were previously described [11]. We used 4 control, XDP, and ΔSVA lines for the outlined experiments.

### Histone extractions

Histone extracts were obtained from XDP and control post-mortem PFC as well as XDP- and control-derived fibroblasts as previously reported [54, 55]. Briefly, tissues and cells were homogenized in 5% Triton buffer on ice and centrifuged at 500 g for 8 min at 4°C to extract nuclei. Then, nuclei were washed twice in 5% Triton buffer and histones were extracted in 0.2 M HCl by vigorous shaking at 4°C for 3 h. After centrifugation, the supernatants containing histones were collected and neutralized with 1 M NaOH. Protein concentrations were determined by Bradford assay.

### Western blots

Western blots from histone extracts were performed using previously described protocols [55]. 10 μg of proteins was resuspended in sample buffer, boiled at 95°C for 5 min, and fractionated on a 10–20% tricine gel for 90 min at 150 V. Next, proteins were transferred to a PVDF membrane in an iBlot Dry Blotting System (Invitrogen, Thermo Fisher, MA), and the membrane was blocked with 5% milk in tris-buffered saline with Tween 20 (TBST) before immunodetection with the following primary antibodies: acetyl-histone H3 (H3K9K14ac_2_; AcH3) (1:500; Millipore, MA; #06–599), and histone H3 (1:500; Millipore, MA; #06–755) overnight at 4°C. Primary antibody incubation was followed by 4 washes (10 min, RT) in TBST before incubation with the secondary antibody for 1h (HRP-conjugated goat anti-rabbit IgG Jackson ImmunoResearch Laboratories, West Grove, PA; #111-035-144). After 4 washes in TBST (10 min, RT) proteins were visualized using the ECL detection system (Thermo Fisher Scientific, MA). Integrated density values (IDV) were measured for each protein band by using an AlphaImager (Protein Simple, CA) gel analyzer and normalized to histone H3.

### Chromatin immunoprecipitation (ChIP)

ChIP for AcH3 was performed as previously described [54, 56]_._ Briefly, proteins were cross-linked to DNA using formaldehyde for 1 h at 37°C and washed three times in a PBS solution containing protease inhibitors at 4°C. Cell pellets were resuspended in 100 μl of 1% SDS lysis buffer supplemented with 10 mM EDTA, 50 mM Tris-HCl (pH 8.1) and protease inhibitors. Next, DNA was sheared to 200–1000 bp using a probe sonicator (Dismembrenator Model 500, Fisher Scientific. PA). Prior to ChIP, a sample of each homogenate representing 1% of the total volume was removed and set aside as input. Samples were diluted with ChIP dilution buffer (0.01% SDS, 1.1% Triton X-100, 1.2 mM EDTA, 16.7 mM Tris-HCl, pH 8.1, 167 mM NaCl) and then incubated in 25 μl of Dynabeads Protein A (Invitrogen, CA) at 4°C overnight with 5 μg of AcH3 (#06–599, Millipore, MA). Dynabeads containing immunoprecipitated samples were separated from supernatant using magnetic columns and washed with 500 μl of the following: a low salt buffer (0.1% SDS, 1% Triton X-100, 2 mM EDTA, 20 mM Tris-HCl, pH 8.1, 150 mM NaCl), a high salt buffer (0.1% SDS, 1% Triton X-100, 2 mM EDTA, 20 mM Tris-HCl, pH 8.1, 500 mM NaCl), a LiCl buffer (0.25 M LiCl, 1% NP-40, 1% deoxycholate, 1 mM EDTA, 10 mM Tris-HCl, pH 8.1), and a TE buffer (10 mM Tris-HCl, 1 mM EDTA, pH 8.0). Cross links were reversed using 2 μl of Proteinase K (New England Biolabs, MA) at 65°C for 2 h. Samples were purified using the UltraClean PCR Cleanup Kit (Mo Bio Labs, CA) based on manufacturer’s protocols. Immunoprecipitated (IP) and input samples were compared to an IgG control as well as a no-antibody mock condition and were only considered to have sufficient DNA for analysis if amount was > 1.5 times mock.

### Quantitative real-time PCR

Genomic DNA pulled down by ChIP as well as in the input samples was analyzed using SYBR Green (Qiagen, Valencia, CA) on an iCycler (Bio-Rad, Hercules, CA), with the following PCR protocol: 50°C for 2 min, 95°C for 10 min, 95°C for 15 sec, 60°C for 1 min for a total of 40 cycles. Each sample was run in duplicates and the average of the duplicate crossing threshold (Ct) values was used in this formula 100*2^(Adjusted input—Ct (IP)) to assess differences in AcH3 association with *TAF1* exons and introns in fibroblasts and NSCs derived from control and XDP patients. The sequences of the primers used for real-time PCR were previously described [6, 15] and are reported in Table 2. Specifically, intron 32-1b, intron 32-2a, intron 32-3c, and intron 32-4c refer to the primers used to amplify the region in intron 32 between exon 32 and the SVA as depicted in Fig 3.

**Table 2:** Primers for *qPCR* amplification.

Primers name	Primers sequences
***TAF1 exon 2***	F: ATCTTCGACTCGTGCTGTCC
	R: GACTTCACCTTCATCATTTACCAA
***TAF1 exon 3***	F: TTTTTCATAGGGTGGGTTAGGA
	R: CCCATCGTCTGCTGGTATCT
***TAF1 exon 16***	F: GGTTTGTTGGGCAGGTTTTT
	R: ACGACCTGTGCGTTTGAAGT
***TAF1 exon 17***	F: TGTCTTTTCCAGGGATGGACT
	R: TGTTCACCTTCAGTCGTTGC
***TAF1 exon 19***	F: CACTGCCCCTTGGAACAC
	R: CTCACCTTCTGCTGTGTTGG
***TAF1 exon 27***	F: GCATTCTGATTTCACATTTCTCC
	R: TGATAGACTCCAAGATGGACGA
***TAF1 exon 32***	F: TCTGAGTGCCTGATTCTTTTCA
	R: ACTGTTGGCCAGAATAAGGTTT
***TAF1 exon 36***	F: CCCAACTGGTCTCATTCAGG
	R: TCACTCCCAGCATCTTCCTC
***TAF1 exon 38***	F: AGACACAAGCTTCAGCAGCA
	R: AAGTCACTGTCCCCAGCAAT
***DSC10***	F: TGTTTGGAATTCAGTGCAAATTTTAT
	R: TGTGCACATGTACCCTAGAAAAA
***DSC12***	F: GATAGGCATGAACCACTGTG
	R: ACCTCAAGAGAGTAGTACAACG
***Intron 32-1b***	F: AGGAAGCAGGGAACTCTCCTA
	R: CTCCCCCACCATTTTCCACT
***Intron 32-2a***	F: ACCAAAGGGAGAAATGGAGAATAGT
	R: AGTCAATGGCACCACCTGTT
***Intron 32-3c***	F: TGTTTGGCTGGAGCAAAGGT
	R: GGCACAATCATTCTCTCGGC
***Intron 32-4c***	F: CTCCCACTCCTGTCTCCCATA
	R: GTGTGCTGAGAGCAGCGTA

### Statistics

Normal distribution of data was not assumed regardless of sample size or variance. For graphical representation display individual values with the central line representing the median, and the edges representing the interquartile range, respectively. Comparison for unrelated samples were performed using a non-parametric Mann Whitney U test and a one-way ANOVA followed by Tukey’s test at a significance level (a) of 0.05. Exact P values are reported.

## Results

### Histone H3 acetylation is not altered in human post-mortem PFC and fibroblasts derived from XDP patients

**Fig 1:**
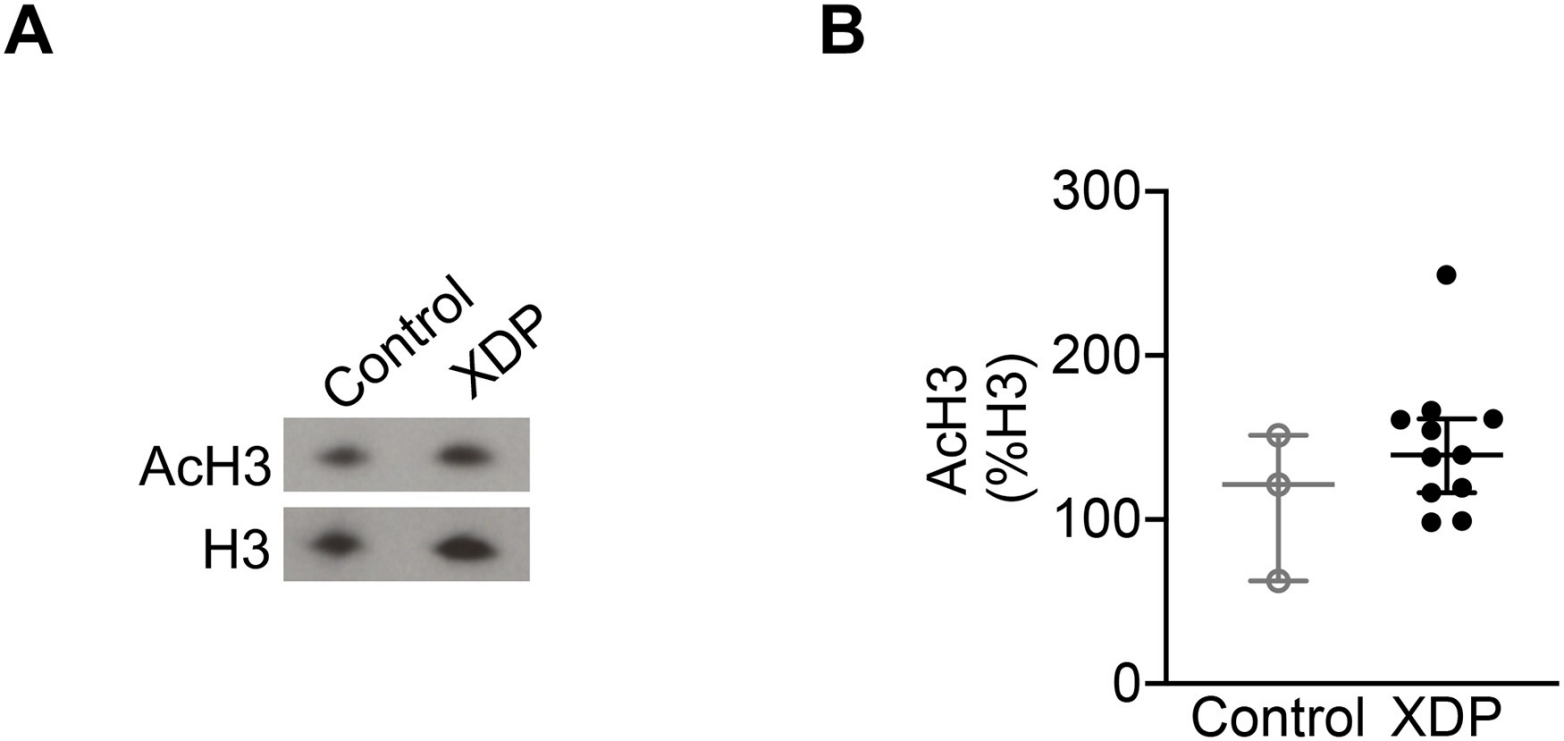
Histone acetylation in XDP post-mortem PFC. The graphs demonstrate the individual integrated density values (IDV) of AcH3 as % of H3 IDV from western blot experiments performed in PFC from XDP (n = 11) and non-neurological control (n = 3), with the central line representing the median, and the edges representing the interquartile range, respectively. **(A)** Representative western blot images of AcH3, and H3 levels. **(B)** AcH3 levels in control and XDP PFC (Mann-Whitney U test = 10, p = 0.1841).

In order to verify whether there were alterations in histone acetylation in the central nervous system (CNS) of XDP patients, total histone H3 acetylation (H3K9K14ac_2_; AcH3) levels were assessed in human post-mortem PFC from XDP (n = 11) and non-neurological controls (n = 3) by western blots (Fig 1A). Our results demonstrate that there were no significant differences in AcH3 levels in XDP PFC compared to controls (Mann-Whitney U test = 10, p = 0.1841) (Fig 1B).

**Fig 2:**
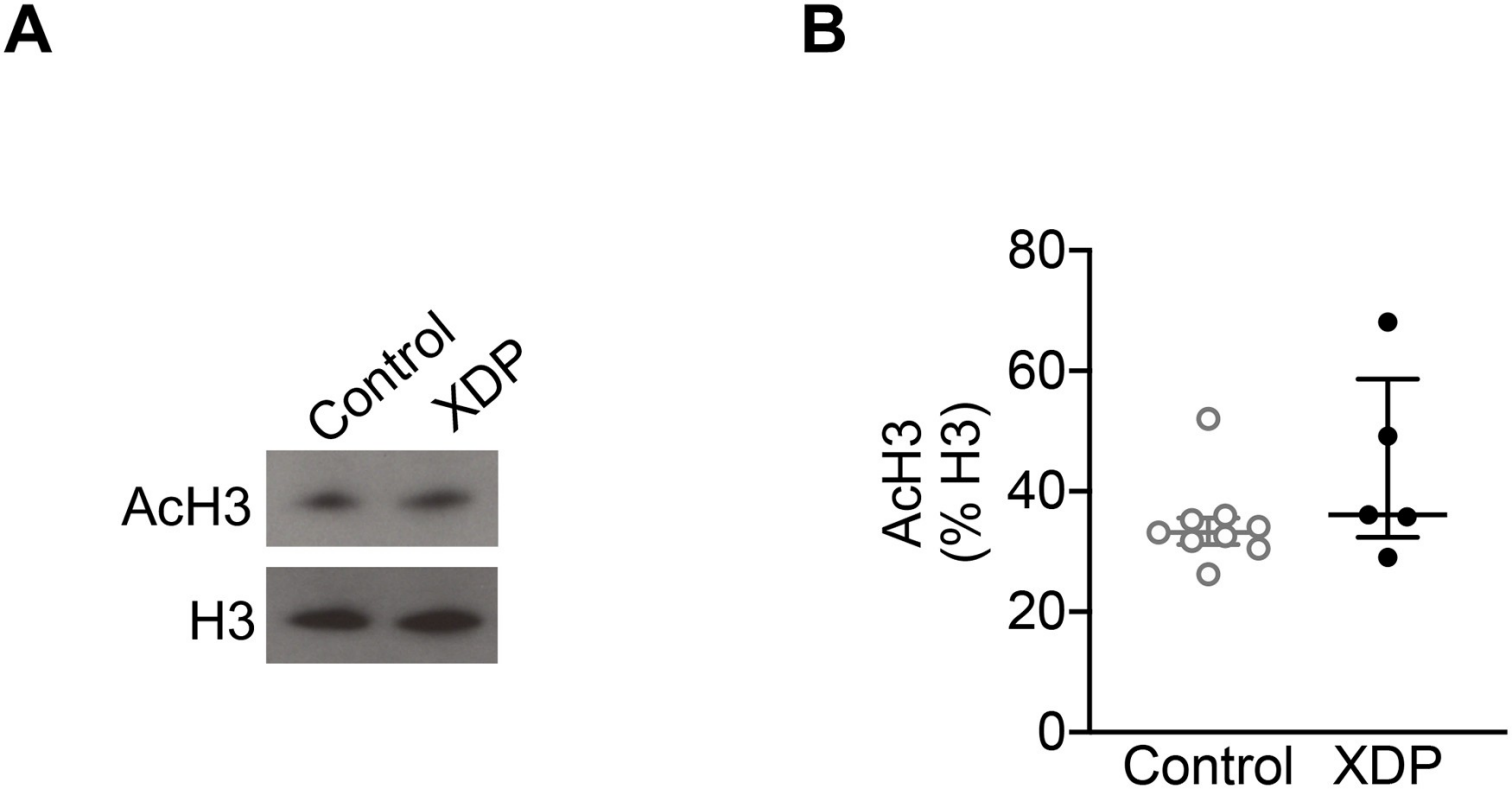
Histone acetylation in XDP-derived fibroblasts. The graphs demonstrate the individual integrated density values (IDV) of AcH3 as % of H3 IDV from western blot experiments performed in fibroblasts from XDP (n = 6) and non-neurological control (n = 10), with the central line representing the median, and the edges representing the interquartile range, respectively. **(A)** Representative western blot images of AcH3, and H3 levels. **(B)** AcH3 levels in control- and XDP-derived fibroblasts (Mann-Whitney U test = 12, p = 0.1898).

Next, we assessed total AcH3 levels in fibroblasts derived from XDP patients (n = 6) and unaffected family members (n = 10) by western blots (Fig 2A). As observed in XDP PFC, there were no significant differences in AcH3 levels in XDP-derived fibroblasts compared to control fibroblasts (Mann-Whitney U test = 12, p = 0.1898) (Fig 2B).

### Acetylated histone H3 association with disease-specific variants along *TAF1* gene is not altered in XDP-derived fibroblasts

**Fig 3:**
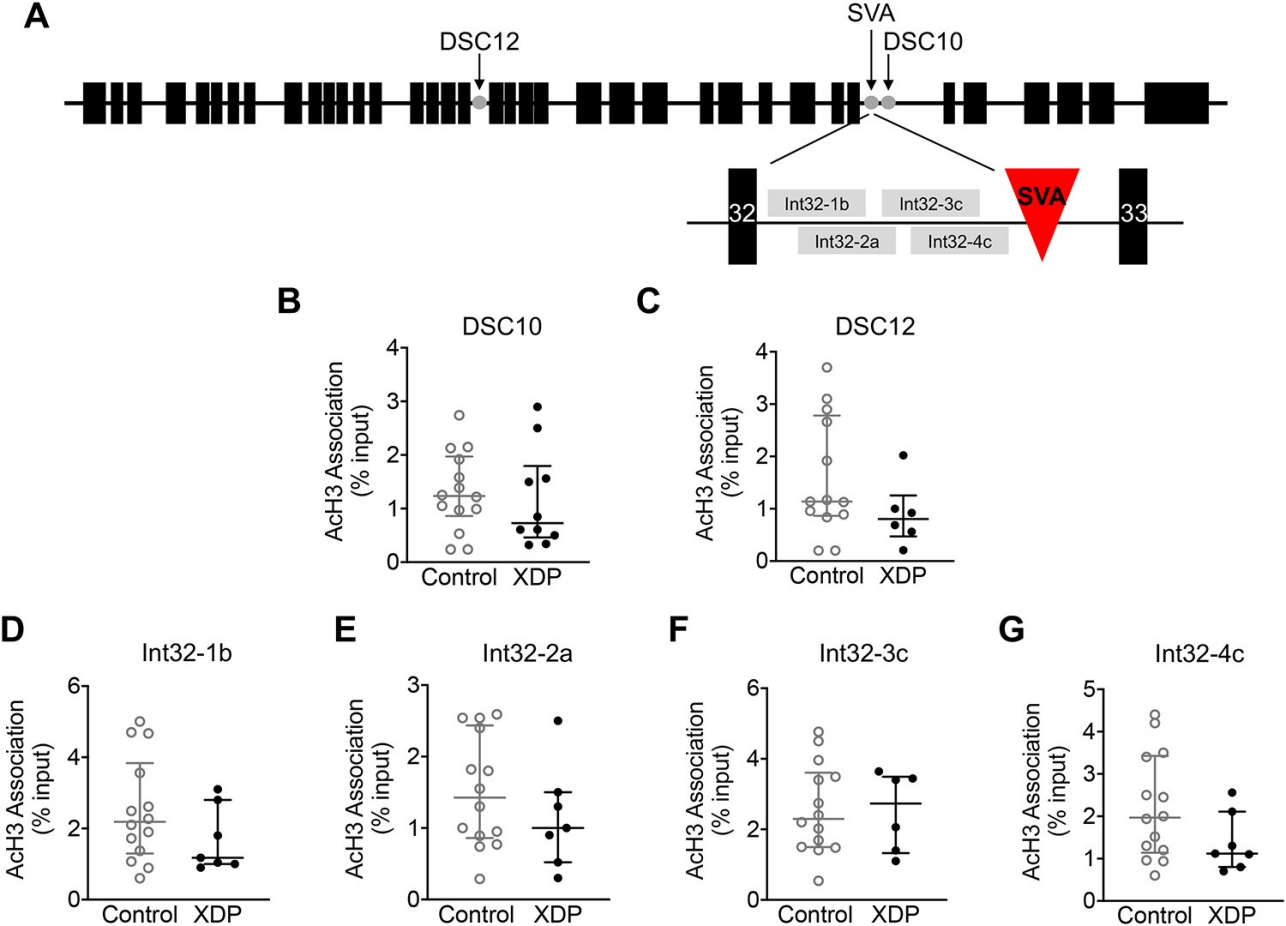
Acetylated histone H3 association with disease-specific variants along *TAF1* gene in XDP-derived fibroblasts. Graphs demonstrate AcH3 association with disease-specific variants measured by ChIP-qPCR, displayed as individual values from control- (n = 14) and XDP-derived fibroblasts (n = 7), with the central line representing the median, and the edges representing the interquartile range, respectively. **(A)** Schematic representation of DSC10, DSC12 and intron 32 along *TAF1* gene. Top schematic: black boxes represent *TAF1* exons, gray dots represent DSC10, DSC12, and SVA. Bottom schematic: black boxes represent intron 32 and intron 33, red triangle represents the SVA, and grey boxes indicate the regions of amplification by intron 32-1b, 2a, 3c, and 4c. **(B)** AcH3 association with DSC10 (Mann Whitney U test = 60, p = 0.5749). **(C)** AcH3 association with DSC12 (Mann Whitney U test = 24, p = 0.2032). **(D)** AcH3 association with intron 32-1b (Mann-Whitney U test = 34, p = 0.2872). **(E)** AcH3 association with intron 32-2a (Mann-Whitney U test = 36, p = 0.3504). **(F)** AcH3 association with intron 32-3c (Mann-Whitney U test = 38.50, p = 0.7930). **(G)** AcH3 association with intron 32-4c (Mann-Whitney U test = 30.50, p = 0.1773).

Given that global histone acetylation levels may not represent the status of AcH3 associated with specific gene loci, we sought to determine whether there were alterations in AcH3 association with *TAF1*. Therefore, we examined sites within exons, the regions of intron 32 surrounding the SVA site at which abnormal splicing/IR occurs, and two additional disease specific variants (DSC10 and DSC12) within the XDP haplotype that are the closest to the SVA insertion (Fig 3A) [5, 6, 11, 57]. First, AcH3 association with DSC10 and DSC12 was assessed using chromatin immunoprecipitation (ChIP) with an anti-AcH3 antibody followed by qPCR in fibroblasts derived from XDP and controls. The results demonstrate that there was no significant change in AcH3 association with DSC10 or DSC12 in XDP-derived fibroblasts (n = 7) compared to control cells (n = 14) (Mann Whitney U test = 60, p = 0.5749, and Mann Whitney U test = 24, p = 0.2032, respectively) (Fig 3B and 3C).

Given that the specific SVA insertion may regulate *TAF1* expression via partial intron retention and aberrant splicing [11], we next assessed AcH3 association with intron 32 across *TAF1* in XDP- and control-derived fibroblasts using ChIP followed by qPCR with a panel of intron 32 specific primers probing the region flanking the SVA (Table 2). The analysis revealed that there was no significant change in AcH3 association with intron 32-1b, intron 32-2a, intron 32-3c, and intron 32-4c in XDP-derived cells (n = 7) compared to control cells (n = 14) (Mann-Whitney U test = 34, p = 0.2872; Mann-Whitney U test = 36, p = 0.3504; Mann-Whitney U test = 38.50, p = 0.7930; and Mann-Whitney U test = 30.50, p = 0.1773, respectively) (Fig 3D–3G).

### Acetylated histone H3 association along the *TAF1* gene is altered in XDP-derived fibroblasts

**Fig 4:**
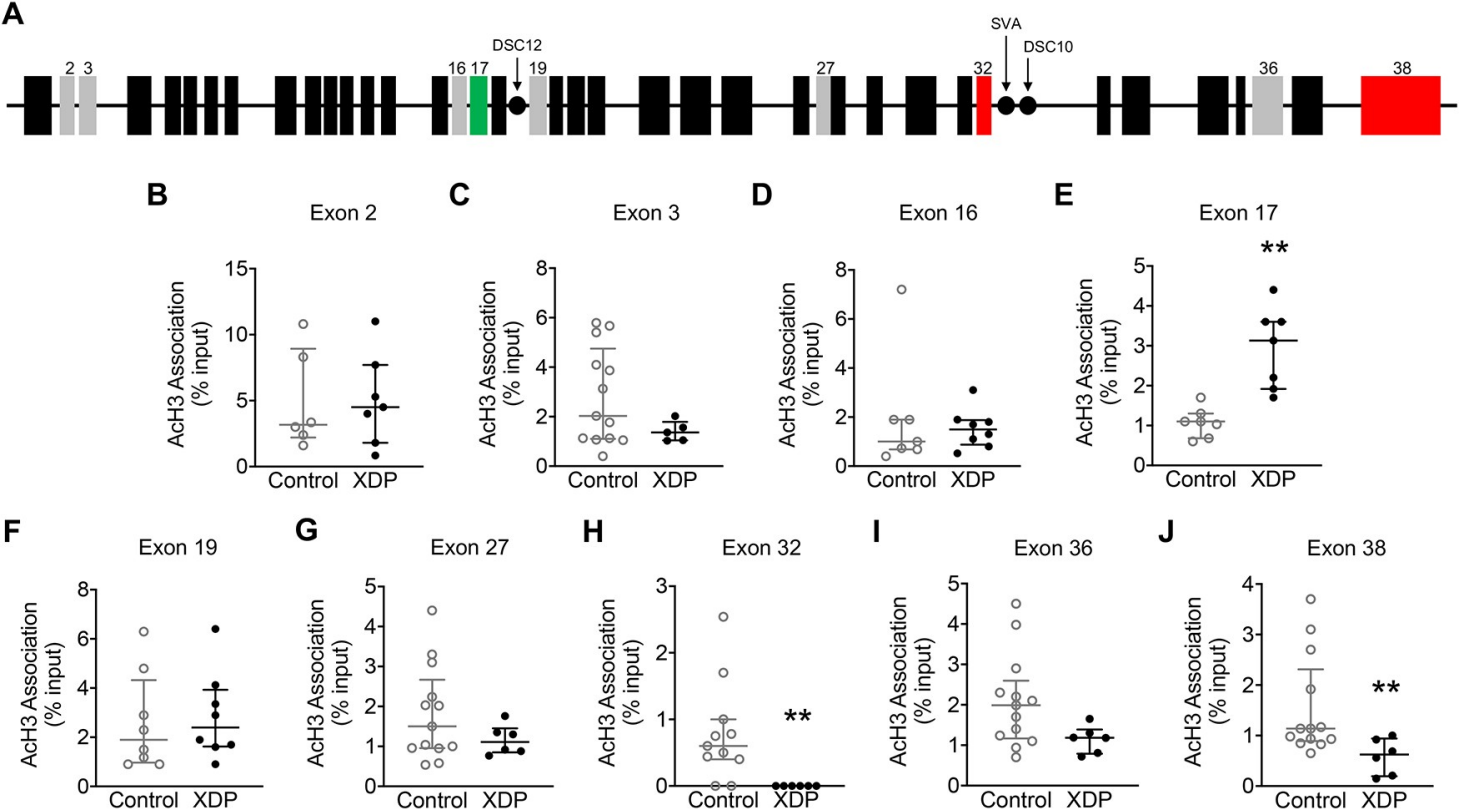
Acetylated histone H3 association with *TAF1* gene exons in XDP-derived fibroblasts. Graphs demonstrate AcH3 association with *TAF1* exons measured by ChIP-qPCR, displayed as individual values from control- (n = 14) and XDP-derived fibroblasts (n = 7), with the central line representing the median, and the edges representing the interquartile range, respectively. **(A)** Schematic representation of *TAF1* gene. Black boxes represent *TAF1* exons; gray boxes represent exons assessed in this study; green boxes represent increased AcH3 association; red boxes represent decreased AcH3 association; black dots represent the disease-specific variants (DSCs) assessed in this study. **(B)** AcH3 association with exon 2 (Mann-Whitney U = 19, p = 0.8357). **(C)** AcH3 association with exon 3 (Mann-Whitney U = 18.50, p = 0.1810) **(D)** AcH3 association with exon 16 (Mann-Whitney U = 25, p = 0.7782) **(E)** AcH3 association with exon 17 (Mann-Whitney U = 0.5000, p = 0.0012). **(F)** AcH3 association with exon 19 (Mann-Whitney U = 25.50, p = 0.5220). **(G)** AcH3 association with exon 27 (Mann-Whitney U = 25, p = 0.2441). **(H)** AcH3 association with exon 32 (Mann-Whitney U = 6, p = 0.0041). **(I)** AcH3 association with exon 36 (Mann-Whitney U = 17, p = 0.0577). **(J)** AcH3 association with exon 38 (Mann-Whitney U = 10, p = 0.0087). ** p < 0.01.

Given that alteration in the transcription of exons surrounding the SVA insertion has been described in XDP [6, 11, 15], we measured AcH3 association across the *TAF1* gene (Fig 4A), interrogating both constitutive and alternative exons that we and others have previously annotated [6, 11, 57], by ChIP with an anti-AcH3 antibody followed by qPCR in fibroblasts derived from XDP patients (n = 7) and unaffected family members (n = 14). The analysis revealed that there were no differences in AcH3 association with exon 2, exon 3, exon 16, exon 19, and exon 27 in XDP-derived fibroblasts compared to controls (Mann-Whitney U = 19, p = 0.8357; Mann-Whitney U = 18.50, p = 0.1810; Mann-Whitney U = 25, p = 0.7782; Mann-Whitney U = 25.50, p = 0.5220; and Mann-Whitney U = 25, p = 0.2441, respectively). Furthermore, there was a trend towards a significant decrease in AcH3 association with exon 36 in XDP-derived fibroblasts compared to controls (Mann-Whitney U = 17, p = 0.0577). Importantly, the analysis revealed a significant increase in AcH3 association with exon 17 as well as a significant decrease at exon 32, and exon 38 in fibroblasts derived from XDP patients compared to fibroblasts derived from unaffected family members (Mann-Whitney U = 0.5000, p = 0.0012; Mann-Whitney U = 6, p = 0.0041; Mann-Whitney U = 10, p = 0.0087, respectively) (Fig 4B–4K).

### Excision of the SVA normalizes deficits in acetylated histone H3 association with *TAF1* exon 32 in XDP-derived NSCs

**Fig 5:**
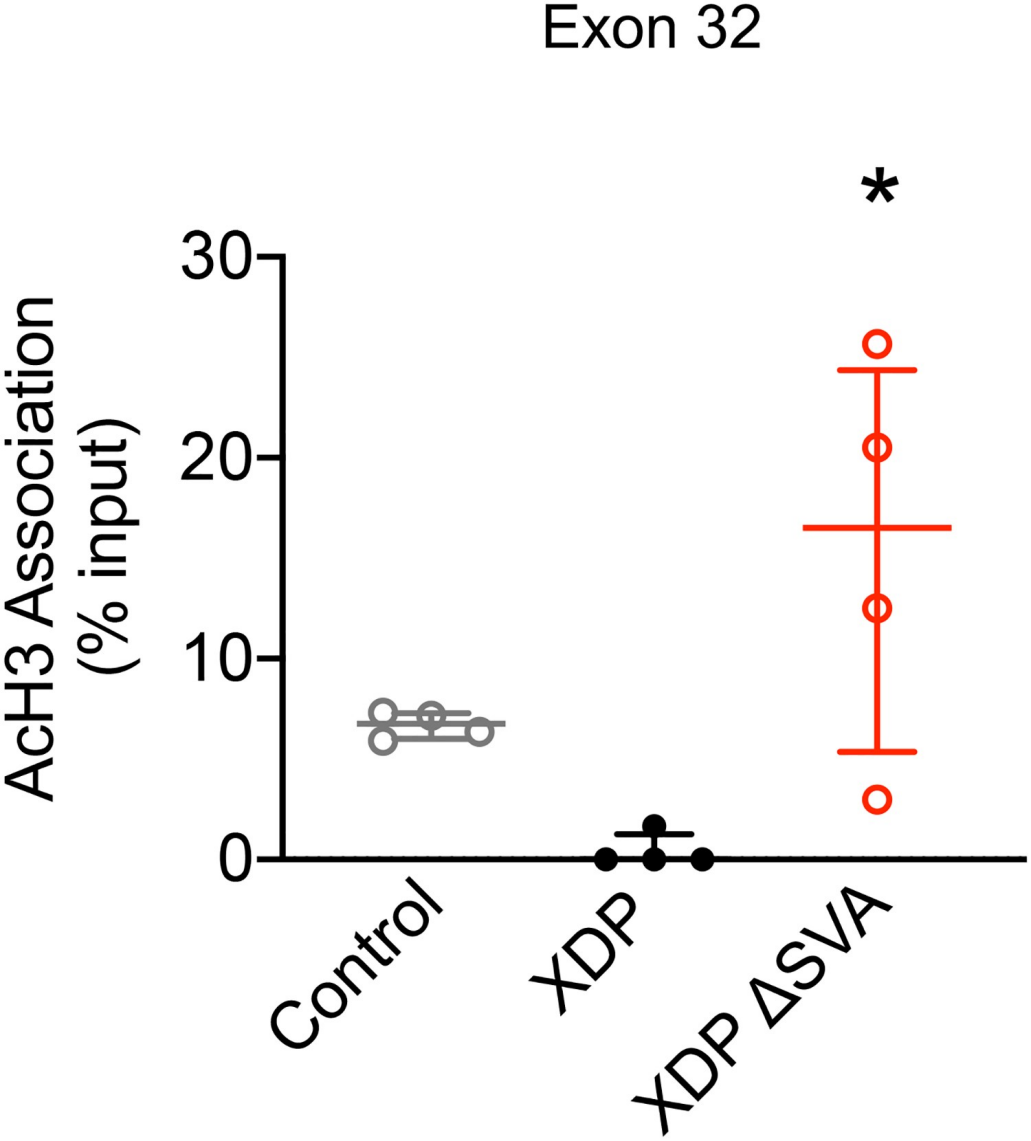
Acetylated histone H3 association with *TAF1* exon 32 in XDP-derived NSCs. Bar graph demonstrates AcH3 association with exon 32 measured by ChIP-qPCR, displayed as individual values from control- (n = 4), XDP- (n = 4), and ΔSVA-derived NSCs (n = 4), with the central line representing the median, and the edges representing the interquartile range, respectively. AcH3 association with *TAF1* exon 32 (one-way ANOVA [F(2, 9) = 6.867, p = 0.0155); Tukey’s test XDP vs control-derived NSCs p = 0.3178; Tukey’s test XDP ΔSVA vs XDP-derived NSCs p = 0.0125). * p < 0.05.

Given that AcH3 association with exon 32, the region adjacent to the SVA-type insertion in the *TAF1* gene, is decreased in XDP-derived fibroblasts, we used NSCs derived from control, XDP and XDP ΔSVA to determine if the presence of the SVA could cause alterations in AcH3 at exon 32 using ChIP followed by qPCR. Our results revealed a significant effect on AcH3 association with exon 32 between XDP-derived NSCs and controls (one-way ANOVA [F(2, 9) = 6.867, p = 0.0155). Importantly, while there were no significant differences in AcH3 association with exon 32 between XDP- and control-derived NSCs, there was a significant increase in AcH3 association with exon 32 in XDP ΔSVA cells compared to XDP-derived NSCs (Tukey’s test, p = 002178, and p = 0.0125, respectively) (Fig 5).

Next, we assessed whether excision of the SVA would alter histone H3 acetylation associated with *TAF1* intron 32 using the same primers as depicted in Fig 3. Our results demonstrated no significant effect on AcH3 association with intron 32-1b as well as no significant difference in AcH3 association with intron 32-1b between XDP- and control-derived NSCs and XDP ΔSVA NSCs (one-way ANOVA [F(2, 9) = 0.2936, p = 0.7525; Tukey’s test, p = 0.9572 and p = 0.7366, respectively) (S1A Fig). Similarly, there was no significant effect on AcH3 association with intron 32-2a, and there was no significant difference in AcH3 association with intron 32-2a in XDP-derived NSCs compared to both control-derived NSCs and XDP ΔSVA NSCs (one-way ANOVA [F(2, 9) = 1.847, p = 0.2128; Tukey’s test, p = 0.3866 and p = 0.8925, respectively) (S1B Fig). Furthermore, the analysis revealed no significant effect of AcH3 association with intron 32-3c and no significant difference in AcH3 association with intron 32-3c in XDP-derived NSCs compared to controls and XDP ΔSVA NSCs (One-way ANOVA [F(2, 9) = 0.7871, p = 0.4842; Tukey’s test, p = 0.9403 and p = 0.4745, respectively) (S1C Fig). Lastly, there was no significant effect of AcH3 association with intron 32-4c as well as no significant difference in AcH3 association with intron 32-4c between XDP- and control-derived NSCs, as well as XDP ΔSVA NSCs (one-way ANOVA [F(2, 9) = 1.238, p = 0.3349; Tukey’s test, p = 0.7509 and p = 0.3055, respectively) (S1D Fig).

## Discussion

In this study, we demonstrated for the first time that total histone H3 acetylation is not altered in human post-mortem PFC or fibroblasts derived from XDP patients. Although previous studies have shown evidence that TAF1 protein may exhibit HAT activity, our results indicate that global AcH3 levels are not affected in XDP brain and cell lines in which *TAF1* expression is reportedly reduced. We did, however, detect local differences in AcH3 association with sites within the *TAF1* gene, albeit only within coding regions. AcH3 association was not altered in XDP fibroblasts at sites within intron 32 flanking the SVA, nor at two disease-specific single nucleotide changes, DSC10 and DSC12, within introns 32 and 18, respectively. Within coding regions, in contrast, there was a significant increase in AcH3 association with exon 17 and a significant decrease in exons 32 and 38 in XDP-derived fibroblasts compared to control-derived cells. Lastly, the excision of the SVA by CRISPR/Cas9-mediated gene editing induced an increase in AcH3 association with *TAF1* exon 32 in XDP ΔSVA NSCs. Together, our findings suggest that the SVA-type insertion alters AcH3 association with specific regions of the *TAF1* gene in XDP.

While decreases in *TAF1* expression are well documented in XDP [6, 11, 12, 14–16], the molecular mechanisms involved in this process remain unknown. Alterations in the epigenome may provide a mechanism where alterations in the chromatin landscape may decrease *TAF1* transcription in XDP. Interestingly, it has been suggested that retroelements, including SVAs, may regulate gene expression [44–51]. Similarly, histone modifications, including methylation, can regulate SVA-type retrotransposon insertions to prevent further retrotransposition or the expression of toxic RNA [26–28]. Furthermore, previous studies have demonstrated that retrotransposons may indirectly alter permissive histone marks such as acetylation by promoting repressive marks such as methylation [29]. Therefore, the expression of retrotransposons, including SVAs, is finely regulated in the genome, a process that is dysregulated during ageing and age-related diseases [58, 59]. However, the exact involvement of histone acetylation in these regulatory mechanisms remain to be clarified. Our findings here demonstrating a decrease in AcH3 association with *TAF1* exon 32 in both fibroblasts and NSCs derived from XDP patients suggest that alterations in histone acetylation in the region flanking the SVA site may be involved in regulating *TAF1* expression in XDP. However, further investigation is required to clarify the role of histone acetylation in regulating SVAs across the genome.

TAF1 activity has also been linked to histone acetylation, specifically, the C-terminal domain was shown to possess HAT activity [30–35], suggesting that a loss of TAF1 activity could lead to a decrease in histone acetylation. Furthermore, the two C-terminal bromodomains on *TAF1* have been shown to selectively bind to and propagate acetylation of histones [30]. Here, we did not detect a significant change in total acetylation of histone H3 in post-mortem PFC or fibroblasts derived from XDP patients. However, we did not assess alterations in histone acetylation in the striatum, the brain region most affected in XDP and characterized by the loss of medium spiny neurons and a decrease in *TAF1* expression [4]. Future studies will assess whether significant alterations in histone acetylation occur in this brain region.

Alterations in global histone acetylation are not always representative of altered acetylation at individual gene promoters. For instance, we previously reported that, while global histone acetylation is not altered in both animal and cellular models of Huntington’s disease (HD), the decrease in AcH3 levels occurs at specific genomic loci and there is a decrease in AcH3 association with specific genes downregulated in HD [54]. Similarly, we previously reported that *TAF1* transcription of exons decreased distal to intron 32 as measured by RNA CApSeq in both fibroblasts and NSCs derived from XDP patients [11]. Specifically, we previously reported a decrease in the expression of *TAF1* transcripts containing exons 32–36 and 38 [6, 15]. In this study, we report that AcH3 association with the *TAF1* gene is altered across this locus in XDP-derived fibroblasts. Specifically, while there was a significant increase in AcH3 association with exon 17, there was a significant decrease in AcH3 association with exon 32, the region flanking the SVA, as well as with exon 38, and a trend towards a significant decrease with exon 36. Collectively, our findings suggest that decreases in AcH3 association with exons 32, 36, and 38 may provide a mechanism whereby these specific *TAF1* transcripts are altered and may contribute to the overall decrease in *TAF1* expression in XDP.

Recently, it has been demonstrated that the excision of the SVA normalized *TAF1* expression in both XDP-derived iPSCs and NSCs [11, 16], and this excision also restored normal splicing in XDP-derived NSCs [11]. Although the exact molecular mechanisms involved in this process are yet unclear, our findings suggest that alterations in histone acetylation may play a role in this process, whereby the presence of the SVA may alter AcH3 levels at this locus in XDP. In addition, although AcH3 association is not altered in XDP-derived NSCs compared to control cells, the excision of the SVA in XDP-derived NSCs was able to increase AcH3 association with exon 32 compared to unedited cells, further suggesting that the SVA is directly linked to altered histone H3 acetylation in XDP. However, genome wide studies, assessing AcH3 association across *TAF1* as well as the entire genome are required to better characterize and determine the role of histone acetylation in XDP pathogenesis.

Lastly, alterations in HAT and HDAC levels and activity have been described in several neurodegenerative diseases, such as HD [43, 60, 61], Alzheimer’s disease (AD) [62, 63], Parkinson’s disease (PD) [64, 65], amyotrophic lateral sclerosis (ALS) [66, 67], and spinal muscle atrophy (SMA) [68]. Therefore, targeting HDACs and HATs has been suggested as potential therapeutic approach for the treatment of neurodegenerative diseases [69–71]. For instance, targeting HDAC2 with the specific inhibitor mithramycin improved neuronal plasticity in cellular model of AD [63], while HDAC6 inhibition improved cognitive decline associated with HD [72], AD [73], tauopathy [74], and Charcot-Marie-Tooth disease [75]. Furthermore, three different HDAC inhibitor clinical trials were approved by the Food and Drug Administration (FDA) for treating neurodegenerative disorders, including sodium phenylbutyrate for the treatment of HD (Clinical Trial NCT00212316) [76] and ALS (Clinical Trial NCT00107770) [66], and valproic acid for the treatment of SMA (Clinical Trial NCT00227266) [68]. Our data suggests that altering AcH3 levels may regulate *TAF1* transcription at least at specific loci, therefore, future studies will assess not only the role of specific HATs and HDACs in order to provide greater mechanistic insight into the pathogenesis of XDP but will also assess the potential therapeutic efficacy of epigenetic inhibitors in XDP-derived cell lines.

## Supporting information

S1 Fig Acetylated histone H3 association with *TAF1* intron 32 in XDP-derived NSCs. Graphs demonstrate AcH3 association with intron 32 measured by ChIP-qPCR, displayed as individual values from control- (n = 4), XDP- (n = 4), and ΔSVA-derived NSCs (n = 4), with the central line representing the median, and the edges representing the interquartile range, respectively. (**A**) AcH3 association with intron 32-1b (one-way ANOVA [F(2, 9) = 0.2936, p = 0.7525; Tukey’s test control vs XDP NSCs p = 0.9572; Tukey’s test XDP vs XDP ΔSVA-derived NSCs p = 0.7366). (**B**) AcH3 association with intron 32-2a (one-way ANOVA [F(2, 9) = 1.847, p = 0.2128); Tukey’s test control vs XDP NSCs p = 0.3866; Tukey’s test XDP vs XDP ΔSVA NSCs p = 0.8925). (**C**) AcH3 association with intron 32-3c (one-way ANOVA [F(2, 9) = 0.7871, p = 0.4842); Tukey’s test control vs XDP NSCs p = 0.9403; Tukey’s test XDP vs XDP ΔSVA NSCs p = 0.4745). (**D**) AcH3 association with intron 32-4c (one-way ANOVA [F(2, 9) = 1.238, p = 0.3349); Tukey’s test control vs XDP NSCs p = 0.7509; Tukey’s test XDP vs XDP ΔSVA NSCs p = 0.3055).
(TIF)

S2 Fig (TIF)

S3 Fig (TIF)

S4 Fig (TIF)

S5 Fig (TIF)

S6 Fig (TIF)
