# Supplementary figures and images for: SVA insertion in X-linked Dystonia Parkinsonism alters histone H3 acetylation associated with TAF1 gene
Source: PLoS One. 2020 Dec 14;15(12):e0243655. doi: 10.1371/journal.pone.0243655 (PMC7735578; doi:10.1371/journal.pone.0243655)

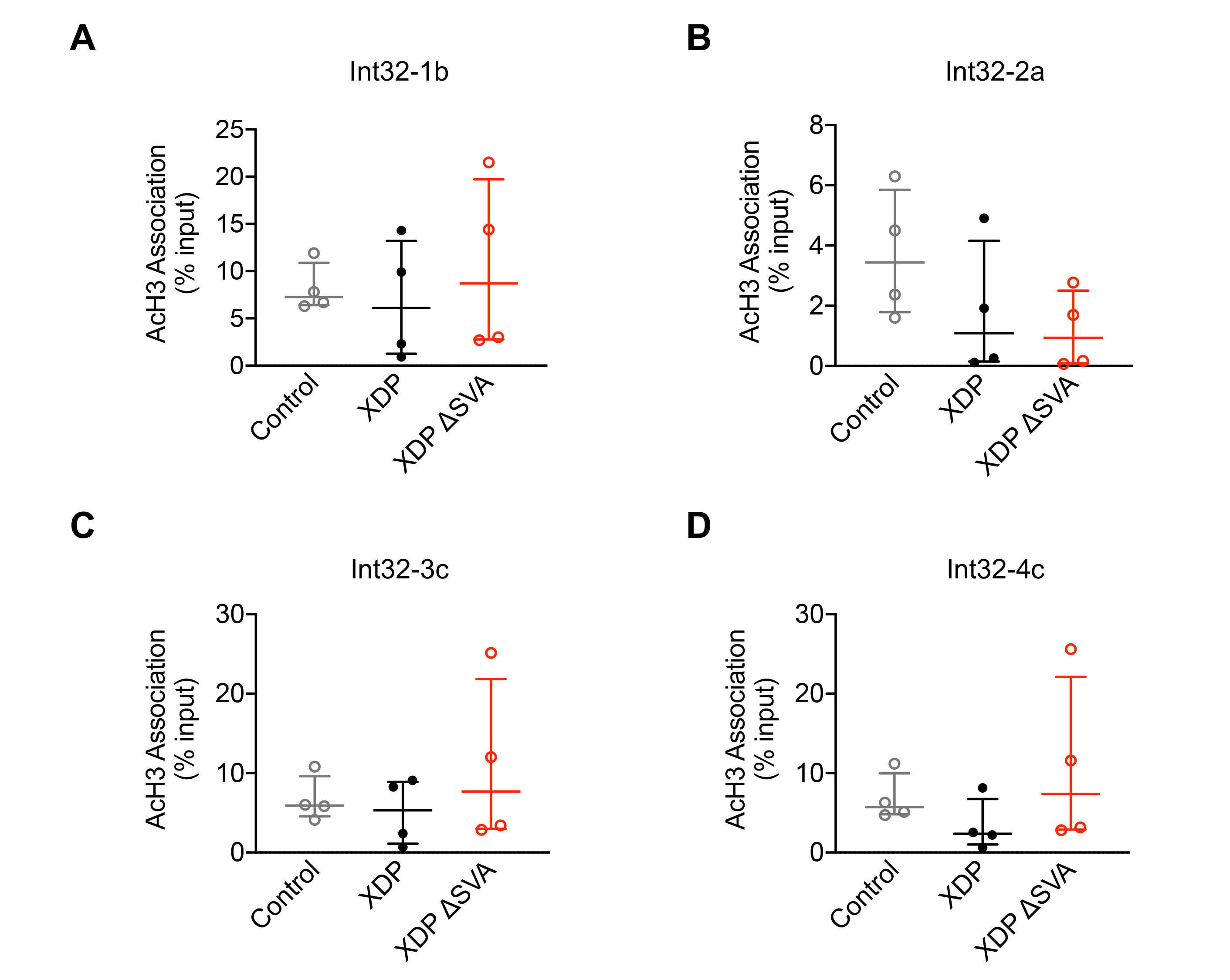

Supplement: S1 Fig — Graphs demonstrate AcH3 association with intron 32 measured by ChIP-qPCR, displayed as individual values from control- (n = 4), XDP- (n = 4), and ΔSVA-derived NSCs (n = 4), with the central line representing the median, and the edges representing the interquartile range, respectively. (A) AcH3 association with intron 32-1b (one-way ANOVA [F(2, 9) = 0.2936, p = 0.7525; Tukey’s test control vs XDP NSCs p = 0.9572; Tukey’s test XDP vs XDP ΔSVA-derived NSCs p = 0.7366). (B) AcH3 association with intron 32-2a (one-way ANOVA [F(2, 9) = 1.847, p = 0.2128); Tukey’s test control vs XDP NSCs p = 0.3866; Tukey’s test XDP vs XDP ΔSVA NSCs p = 0.8925). (C) AcH3 association with intron 32-3c (one-way ANOVA [F(2, 9) = 0.7871, p = 0.4842); Tukey’s test control vs XDP NSCs p = 0.9403; Tukey’s test XDP vs XDP ΔSVA NSCs p = 0.4745). (D) AcH3 association with intron 32-4c (one-way ANOVA [F(2, 9) = 1.238, p = 0.3349); Tukey’s test control vs XDP NSCs p = 0.7509; Tukey’s test XDP vs XDP ΔSVA NSCs p = 0.3055). (TIF) [file pone.0243655.s001.tif]

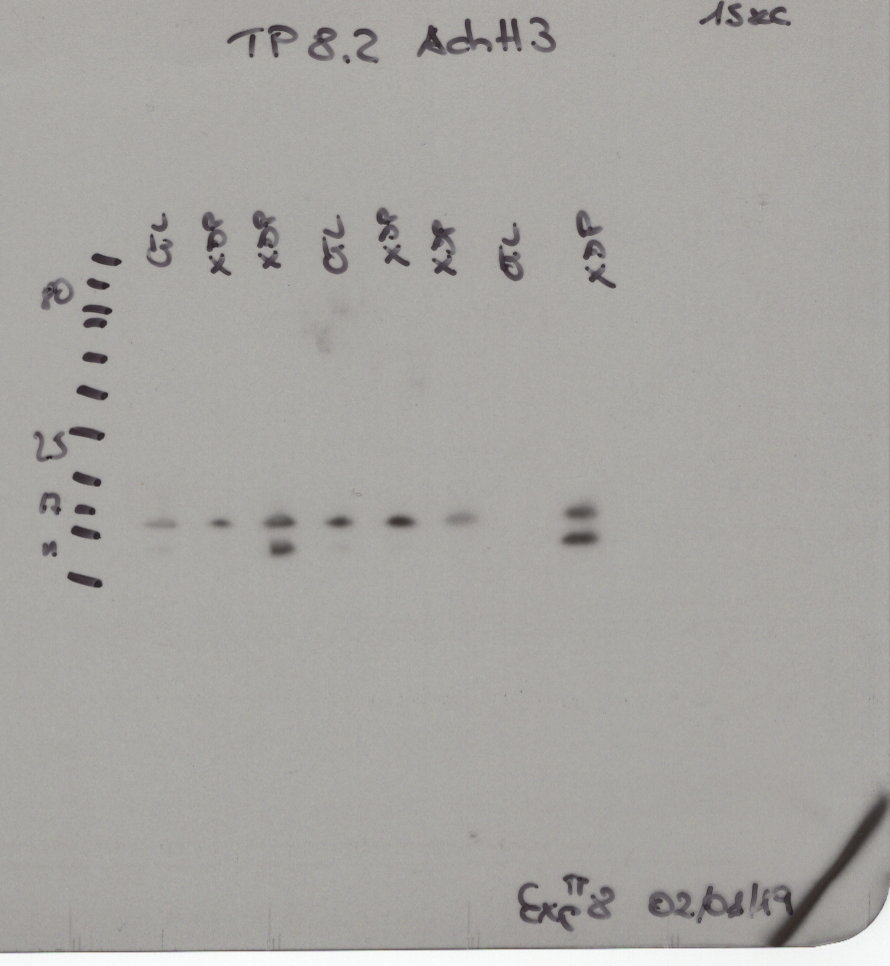

Supplement: S2 Fig — (TIF) [file pone.0243655.s002.tif]

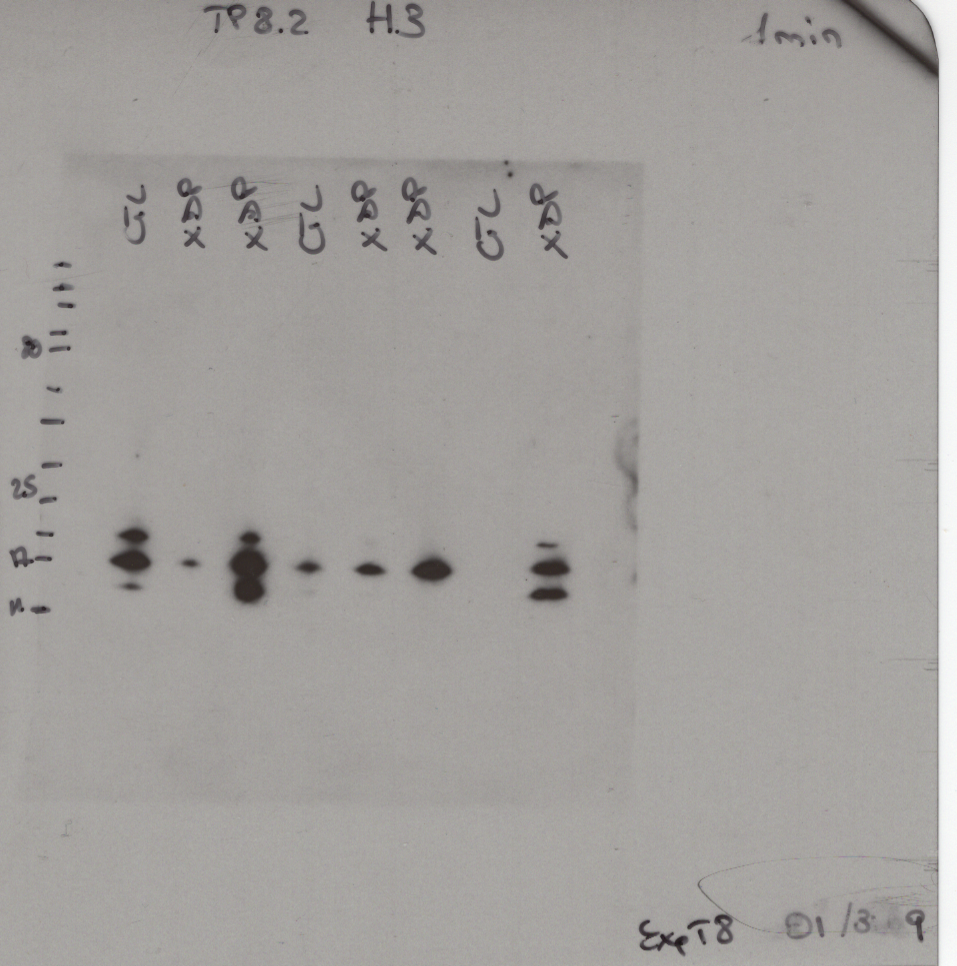

Supplement: S3 Fig — (TIF) [file pone.0243655.s003.tif]

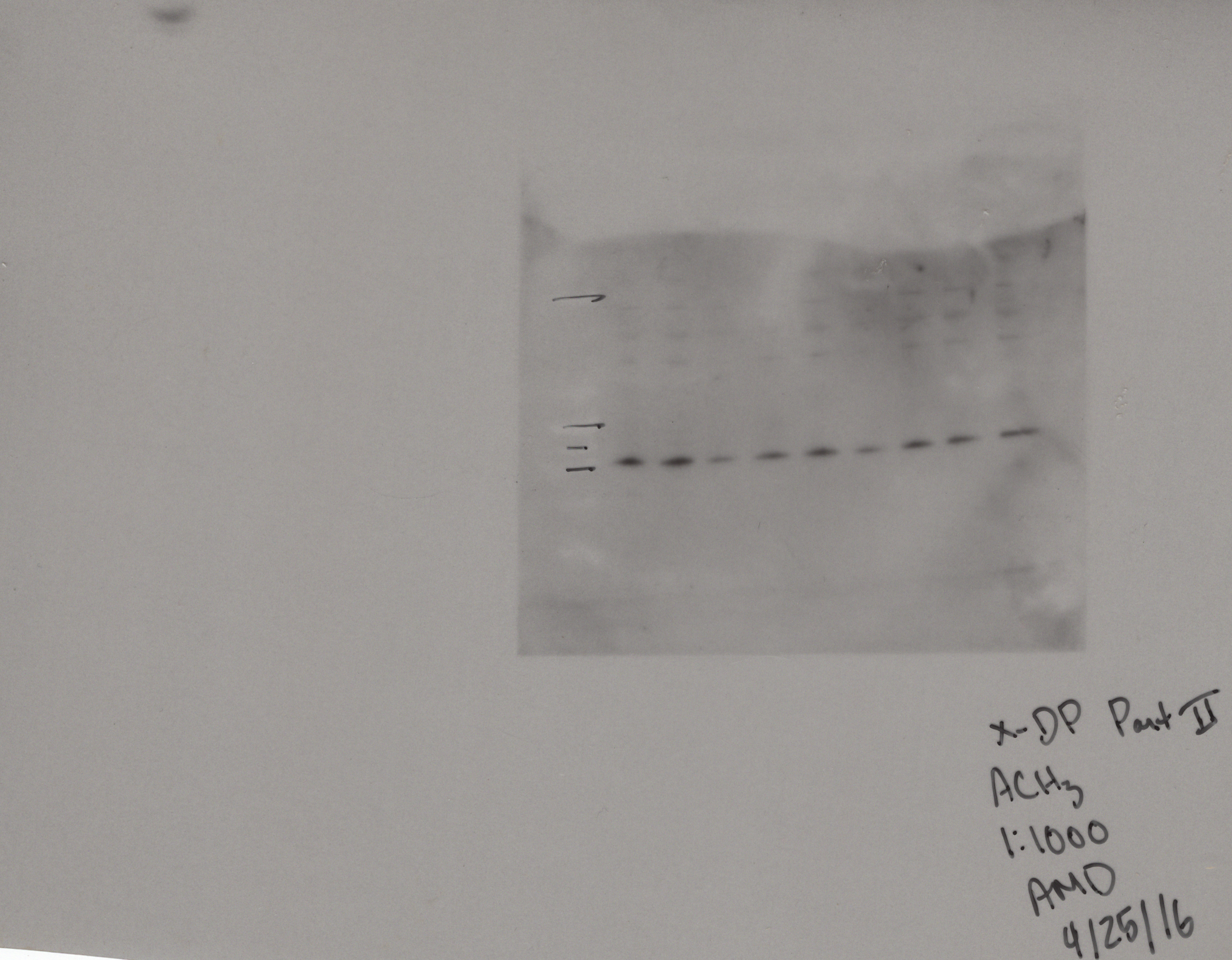

Supplement: S4 Fig — (TIF) [file pone.0243655.s004.tif]

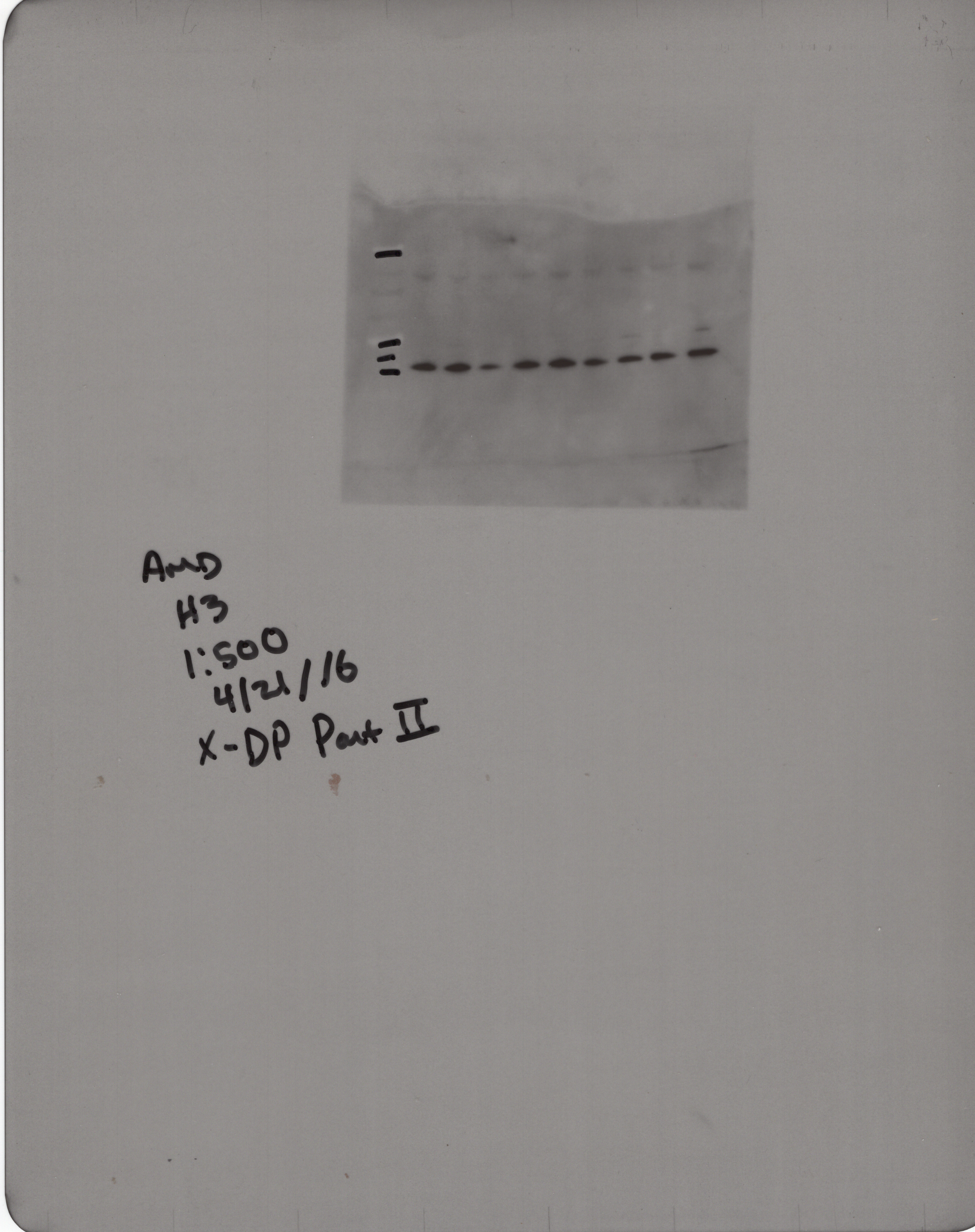

Supplement: S5 Fig — (TIF) [file pone.0243655.s005.tif]

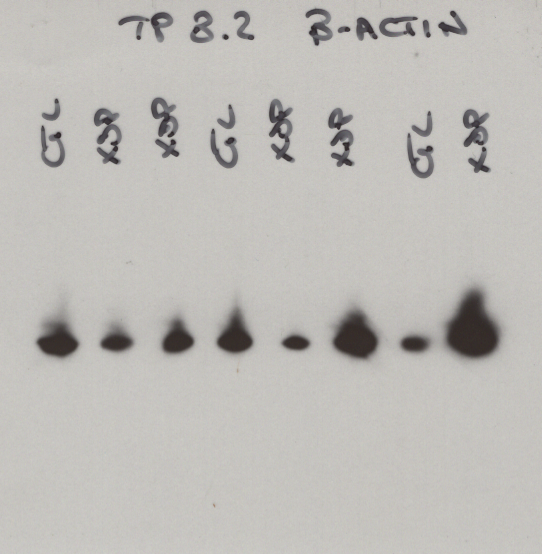

Supplement: S6 Fig — (TIF) [file pone.0243655.s006.tif]
